# Comparative Effects of Different Dosages of hCG on Follicular Development in Postpartum Dairy Cows With Cystic Ovarian Follicles

**DOI:** 10.3389/fvets.2018.00130

**Published:** 2018-06-29

**Authors:** Tetsushi Ono, Mitsuhiro Takagi, Chiho Kawashima, Missaka P. B. Wijayagunawardane, Peter L. A. M. Vos, Masayasu Taniguchi, Fuminori Tanihara, Takeshige Otoi

**Affiliations:** ^1^Faculty of Veterinary Medicine, Okayama University of Science, Imabari, Japan; ^2^Laboratory of Theriogenology, Joint Faculty of Veterinary Medicine, Yamaguchi University, Yamaguchi, Japan; ^3^Field Center of Animal Science, Obihiro University of Agriculture and Veterinary Medicine, Obihiro, Japan; ^4^Department of Animal Science, University of Peradeniya, Peradeniya, Sri Lanka; ^5^Department of Farm Animal Health, Faculty of Veterinary Medicine, Utrecht University, Utrecht, Netherlands; ^6^Laboratory of Animal Reproduction, Faculty of Bioscience and Bioindustry (Ishii campus), Tokushima University, Tokushima, Japan

**Keywords:** dairy cow, cystic ovarian follicles, hCG treatment, ovarian follicular development, postpartum

## Abstract

The objective of this study was to determine the effects of different intramuscular dosages of human chorionic gonadotropin (hCG) on ovarian follicular development of dairy cows diagnosed with refractory cystic ovarian follicles (COFs). Cows diagnosed with COFs (≥25 mm in diameter) were allocated to four treatment groups: hCG-1 (*n* = 3), a single dose of 4,500 IU on day 1; hCG-2 (*n* = 3), 2,250 IU on days 1 and 3; hCG-3 (*n* = 3), 1,500 IU on days 1, 3, and 5; and hCG-C (*n* = 3) received saline on day 1. Blood sampling and ovarian ultrasonographic (US) examinations were performed on days 1, 3, 5, 7, and 14. A progesterone (P4) value < 1 ng/ml was used as an indicator of absence of a functional CL. A significant increase (*P* < 0.05) in the number of follicles < 4 mm in diameter was observed in the hCG-2 group on day 5. Additionally, there was a significant difference in the number of follicles < 4 mm (*P* < 0.05) between both the hCG-2 and hCG-3 groups compared to the hCG-C group on day 5, and a tendency (*P* = 0.08) toward a difference in the number of 5–9 mm follicles in groups hCG-3, hCG-2, and hCG-1, compared with the hCG-C group on day 7. The proportion of cows on days 7 and 14 with P4 > 1 ng/ml was 100% (3/3) and 100% (3/3) in group hCG-1; 100% (3/3) and 67% (2/3) in group hCG-2; 67% (2/3) and 100% (3/3) in group hCG-3; and 33% (1/3) and 33% (1/3) in group hCG-C, respectively. Strong tendencies of P4 increases in group hCG-1 (*P* = 0.054) and hCG-2 (*P* = 0.051) were measured after hCG administration. Additionally, P4 values tended to be higher (*P* = 0.07) for group hCG-1 compared to group hCG-C on day 5. The preliminary findings of this study suggest that multiple smaller doses of hCG might be equally effective as a single large dose of hCG in modulating ovarian follicular development in dairy cows with COFs.

## Introduction

Cystic ovarian follicles (COFs) are a common cause of reproductive failure in female cattle (1–4). Traditionally, COFs were defined as non-ovulatory follicular structures measuring greater than or equal to 25 mm that persist for at least 10 days without the presence of a functional corpus luteum (CL) (4, 5). This definition has been modified to include follicular structures with diameters ranging from 17 to 22 mm and persisting for 7 to 8 days (3, 4). Previous reports estimate that the incidence of COFs in dairy cattle varies between 6 and 60%. They may spontaneously resolve before the first postpartum ovulation and thus may go undetected (1, 2). Cystic ovarian follicles are thought to result from neuro-endocrinological dysfunction of the hypothalamic-pituitary-gonadal axis (2, 6), often combined with local dysregulation during follicular development (7). However, despite extensive research, the mechanism of COF development is unclear (7–9).

Under field conditions, the diagnosis of COFs is based on behavioral abnormalities, transrectal examination of the genital tract including ultrasonography (US), and occasionally, blood progesterone (P4) profiles. Different treatment regimens involving gonadotropin releasing hormone (GnRH), human chorionic gonadotropin (hCG), prostaglandin F2α (PGF2α), and P4 have been used with variable ovarian responses and cure rates (2, 10, 11). Human chorionic gonadotropin has been used to successfully treat COFs non-responsive to GnRH (2, 5). However, due to its high molecular weight and carbohydrate content, repeated administration of hCG may result in the development of hCG antibodies in cattle (12, 13). Regarding this point, Giordano et al. (13) reported that circulating levels of hCG antibody that may interfere with ovulation were not reached until 7 to 14 days after the hCG (2,000 IU) injection. These results suggest that low doses of hCG administered more than once within 7 days may be a practical alternative for the treatment of COFs in dairy cows. However, to our knowledge, administration of a single dose of 3,000 to 10,000 IU hCG is routinely used to treat COFs in cattle, and no field studies have explored the administration of repeated low doses of hCG in cows with COFs, especially focusing on ovarian follicular development after the hCG administration. Therefore, determining the therapeutic significance of hCG for COFs that are non-responsive to GnRH is important. The objective of this preliminary field study was to explore the effects of different hCG dosages (single vs. multiple divided doses) on ovarian follicular development in dairy cows with COFs.

## Materials and methods

### Animals and management

Lactating Holstein cows from five commercial dairy farms in Yamaguchi Prefecture, Japan, were used for this study. The study was performed from April 2013 to November 2014. All animals were cared for according to the Guide for the Care and Use of laboratory Animals (Faculty of Veterinary Medicine, Yamaguchi University, Yamaguchi, Japan), and informed consent was obtained from all the dairy farmers. Cows were housed in free stall barns and fed *ad libitum* total mixed ration diet and water. The diet was formulated to meet the requirements of high-production lactating dairy cows (NRC recommendations). The body condition scores of the cows were between 3.25 and 3.5, and the average yearly herd milk yield was between 8,000 and 9,000 kg. Throughout the experiments, cows were milked twice daily, and concurrent systemic or metabolic diseases were monitored. All cows used in the present study were free of these diseases.

### Diagnosis/detection of cows with cystic ovarian follicles, treatment, and blood sampling

Cows were selected during routine farm visits based on the farmers' complaints and postpartum reproductive records provided by local veterinarians. Based on preliminary selection by the veterinarians, postpartum cows with an anovulatory follicle greater than 25 mm in diameter by rectal palpation and US monitoring that persisted on the ovary for more than 7 days without a functional CL, were diagnosed with COFs in accordance with published reports (2–4), by one veterinarian. Among the cows with COFs, some had been artificial inseminated more than three times and had not become pregnant, and had received additional hormonal treatment (excluding hCG) during the postpartum period. In this study, plasma P4 concentrations of all stored collected blood samples were measured following treatment with one assay. Therefore, the function of the CLs of the cows in each treatment group was unknown at the time of the first hCG administration. Based on the P4 concentrations measured after hCG administration, all the cows with a P4 concentration of more than 1.0 ng/ml at the commencement of the study were considered to have a functional CL (14), and excluded from the study.

Cows diagnosed with COFs were randomly allocated to four treatment groups; hCG-1 (*n* = 3; age: 2.3 ± 0.6 years), a single dose of 4,500 IU on day 1 (first day of treatment); hCG-2 (*n* = 3; age: 4.0 ± 2.6 years), 2,250 IU on days 1 and 3; hCG-3 (*n* = 3; age: 5.3 ± 2.1 years), 1,500 IU on days 1, 3, and 5; and the control, hCG-C (*n* = 3; age: 3.3 ± 1.5 years), a single saline dose (1 ml) on day 1. All injections were intramuscular. Treatment began for hCG-1 at 149.0 ± 19.9 days in milk (range; 126–161 days); for hCG-2 at 177.0 ± 157.2 (range; 67–357 days); for hCG-3 at 221.0 ± 124.2 (range; 90–337 days); and hCG-C at 113.3 ± 75.4 (range; 63–200 days).

**Figure 1:**
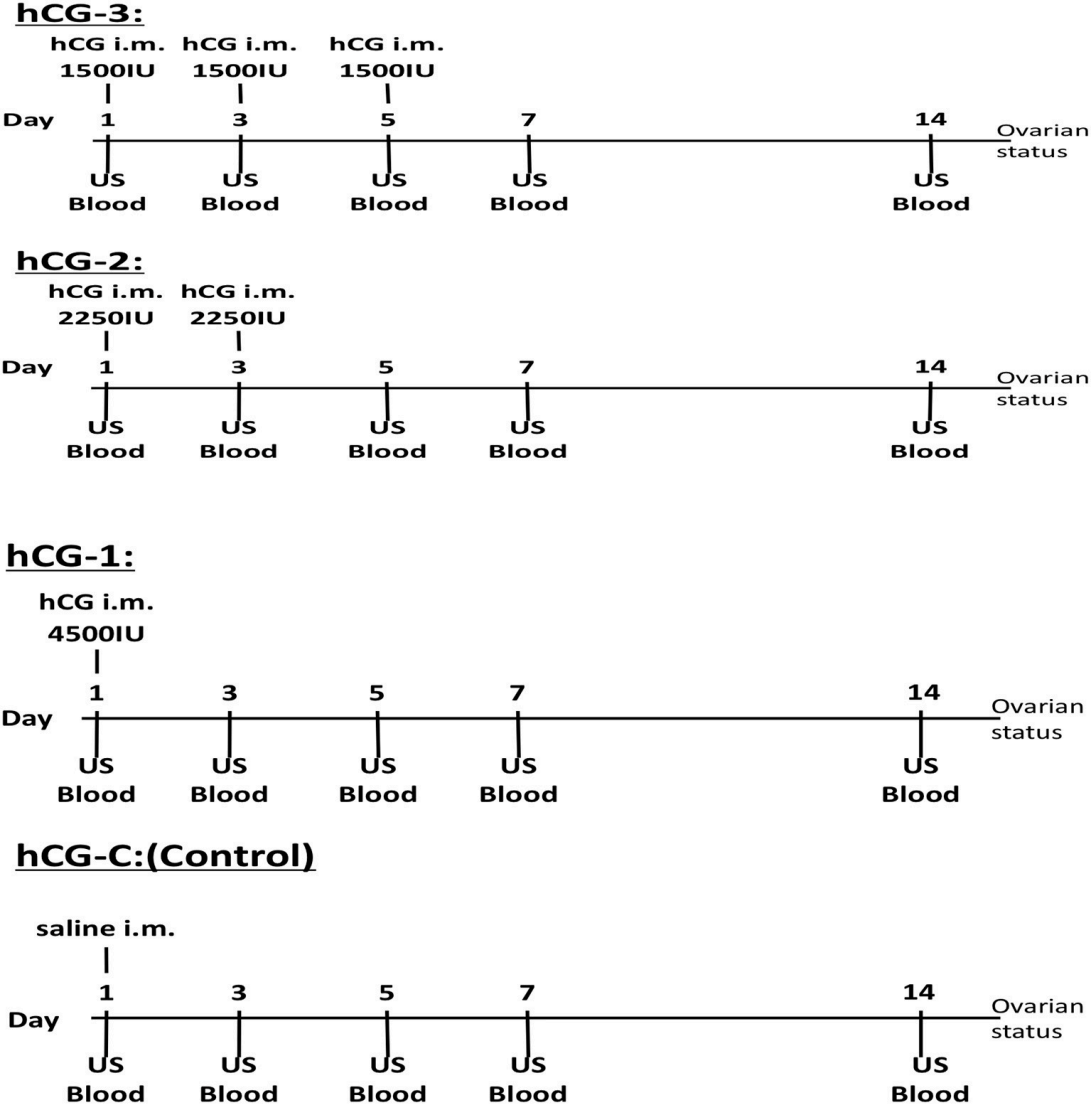
Experimental protocol of the hCG administrations, blood sampling, and ovarian ultrasonographic examination for each group.

Jugular vein blood samples from each group were collected for serum P4 profiling, on days 1, 3, 5, 7, and 14. Collected blood samples were immediately placed in an icebox and transported to the laboratory. After centrifugation at 500 × g for 10 min at room temperature, the serum was separated and frozen at −30°C until hormone analysis. Transrectal US scanning of both ovaries was also conducted on days 1, 3, 5, 7, and 14 using a linear array US scanner equipped with a 5-MHz rectal probe for evaluating follicular dynamics. At each sampling point, ovarian structures were evaluated by US scanning and video recorded; both the number and size of visible antral follicles and CLs were measured. The study procedures, including hCG administration, blood sampling, and US monitoring, are illustrated in Figure 1.

### P4 assay

Plasma P4 concentrations were determined by an enzyme immunoassay after extraction with a diethyl ether, as previously described (15). The extraction efficiency of P4 was 90%. The 50% effective dose of the P4 assay was 0.66 ng/ml, and the minimum detectable concentration of this assay was 0.05 ng/ml. The mean intra-assay and inter-assay coefficients of variation were 2.9 and 1.9%, respectively. Hormonal data were used to confirm and support the US monitoring of CL and follicular development.

### Clinical data analysis

After hCG administrations, the occurrence of ovulation was detected by US monitoring, and CL formation was confirmed through plasma P4 concentrations higher than 1 ng/ml in three consecutive samples. The number of follicles was counted and categorized as follows: less than 4, 5–9, 10–14, and 15–19 mm.

### Statistical analysis

All statistical analyses were carried out using StatView® computer software (Abac Concept, Inc., Berkeley, CA, USA). The area of COF, number of follicles, and P4 concentrations were expressed as means ± SEM. A non-parametric analysis of variance (Kruskal-Wallis) was used to explore the between-group (treatment) differences in follicular number and the P4 concentrations, and the within-group (each day after hCG administration) differences in P4 concentrations. Probability values less than 0.05 (*P* < 0.05) were considered statistically significant, while *P*-value less than 0.1 (*P* < 0.1) were considered to indicate a trend toward significance.

## Results

### Ovarian activity after hCG administrations detected by US

Table 1 provides a summary of ovarian activity of the four treatment groups during the 14-day experimental period. With the exception of one cow in the hCG-2 group, all animals with COFs exhibited size reductions, luteinization, or ovulation after hCG administration. After hCG administration, new CL development was observed in 3, 3, 3, and 1 cow(s) out of 3 cows each in the hCG-1, hCG-2, hCG-3, and hCG-C groups, respectively. On day 14, new COFs were not observed in group hCG-1 or group hCG-2, but one was identified in a cow in group hCG-3.

**Table 1:** Summary of the ovarian activities of four groups during the experimental periods.

**hCG treatment group**		**hCG-1**	**hCG-2**	**hCG-3**	**hCG-C**
**Number of Cows**		**3**	**3**	**3**	**3**
**Age, years**		**2.3 ± 0.6**	**4.0 ± 2.6**	**5.3 ± 2.1**	**3.3 ± 1.5**
Cystic ovarian follicle (≥25 mm)	Area increased	0	1	0	0
	Area reduced	3	2	3	3
	Luteinized	1	0	0	0
	Ovulation	0	0	1	0
Appearance of new CL	Number	3	3	3	1
Appearance of new CL	Day 3	1	1	0	0
	Day 5	1	0	1	0
	Day 7	1	2	2	0
	Day 14	0	0	0	1
Appearance of new cystic ovarian follicle	Day 14	0	0	1	1

*Cows diagnosed with cystic ovarian follicles were allocated into four treatment groups; hCG-1, a single dose of 4,500 IU on day 1; hCG-2, 2,250 IU on days 1 and 3; hCG-3, 1,500 IU on days 1, 3, and 5; and control, hCG-C received saline on day 1*.

**Figure 2:**
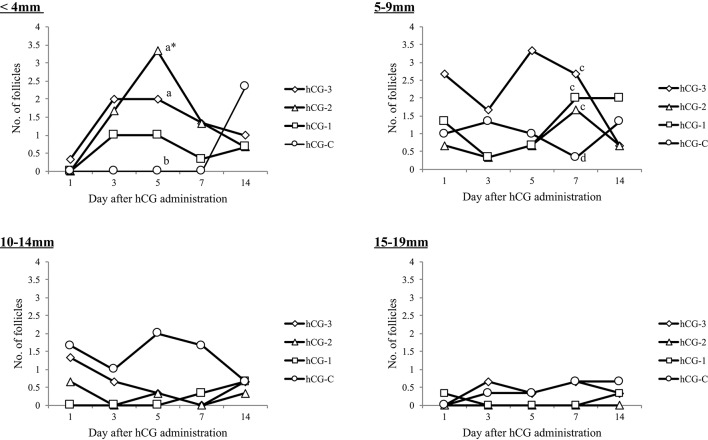
Mean number of developed follicles, categorized by diameter, in each group. ^*^Indicates that the number of follicles on day 5 in the hCG-2 group was significantly different than other days within the group; a,b, significantly different (*P* < 0.05); c,d, tendency for a difference (*P* < 0.1).

Figure 2 shows the mean number of follicles classified by diameter. A significant increase in the number of follicles < 4 mm in diameter (*P* < 0.05) was observed in group hCG-2 on day 5 compared to the other days. Additionally, a significant difference in the number of follicle < 4 mm (*P* < 0.05) between both groups hCG-2 and hCG-3, compared with group hCG-C, was observed at day 5. A tendency (*P* = 0.08) for the number of follicles 5–9 mm to increase in groups hCG-3, hCG-2, and hCG-1 compared with group hCG-C on day 7 was observed. No differences were observed between groups for number of follicles that were 10–14 or 15–19 mm in diameter.

**Figure 3:**
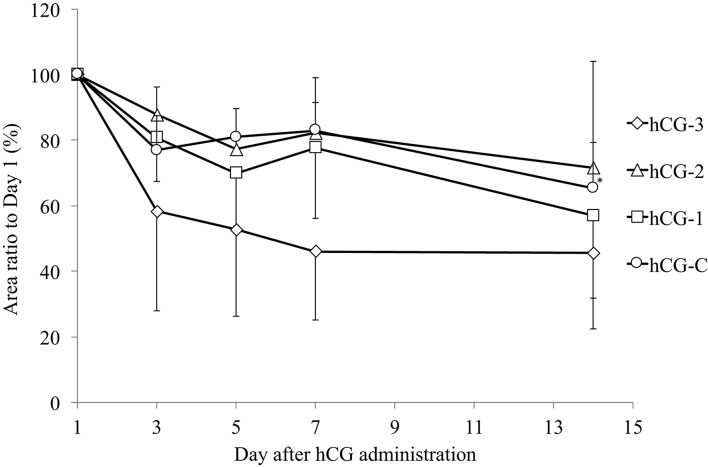
Mean (± SEM) change in the area of cystic ovarian follicles in cows in each group. The area of the cystic follicle on day 1 was considered 100%. ^*^Indicates significant reduction in cystic ovarian follicle area compared with day 1.

Figure 3 shows the change in COF area after the hCG administration in each group, compared to the original COFs area. Although the size of all COFs from all groups decreased, the only significant reduction in area was observed in the hCG-C group.

### Progesterone concentration after hCG administration

**Figure 4:**
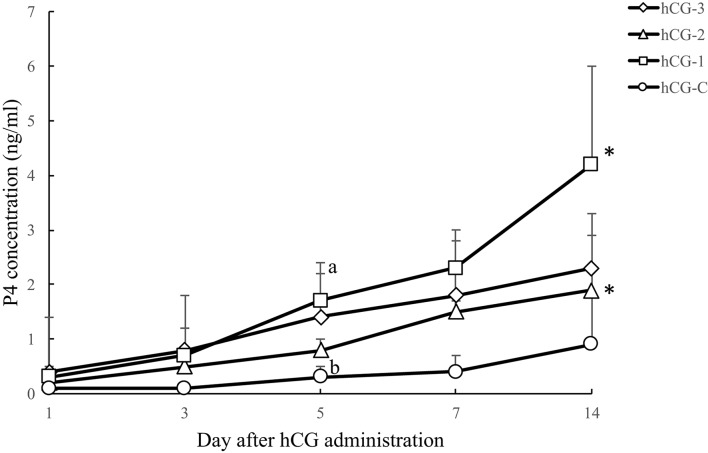
Serum P4 concentration (mean ± SEM) of each hCG group. a,b, a tendency (*P* = 0.07) for a difference between the hCG-1 and hCG-C groups was observed. ^*^A tendency for an increase in P4 concentrations was observed in the hCG-1 (*P* = 0.054) and hCG-2 (*P* = 0.051) groups at day 14 compared with day 1.

Figure 4 shows the blood P4 profiles of each group after hCG administrations. A tendency for the P4 concentration to increase (*P* < 0.1) was observed in groups hCG-1 and hCG-2 on day 14 compared with day 1. Progesterone levels in all the hCG groups on day 14 were greater than 1 ng/ml, suggesting the development of a functional CL. The proportion of cows on day 7 and 14 with P4 > 1 ng/ml was 100% (3/3) and 100% (3/3) in hCG-1; 100% (3/3) and 67% (2/3) in hCG-2; 67% (2/3) and 100% (3/3) in hCG-3; and 33% (1/3) and 33% (1/3) in hCG-C, respectively. Additionally, a tendency for a difference in the P4 concentration (*P* = 0.07) was observed between groups hCG-1 and hCG-C on day 5.

## Discussion

In the present study, we focused on the effectiveness of alternative methods of administration of hCG, i.e., single or multiple divided doses for cows diagnosed with refractory COFs, to assess ovarian responses and serum P4 concentrations. Although the number of cows included in the study was small, our results suggest that repeated low doses of hCG over time may enhance the ovarian follicular development of the cows with COFs.

Different responses of COFs to hCG treatment have been reported, including partial or complete luteinization, regression, and ovulation (1, 2). In the present study, a significant regression of the refractory COFs was observed in the hCG-3 group on day 7 compared to the other groups, indicating that the repeated administration of low dose hCG may be more effective in regressing COFs. A dose of 3,000 IU hCG resulted in the development and selection of new dominant follicles 4 days after treatment (11). The number of follicles < 4 mm in diameter increased after administration of hCG in groups hCG-2 and hCG-3. This may be the result of the low dose and successive administration of hCG. The follicles may have increased in size to 5–9 mm in diameter by day 5 or day 7; however, our observation that the number of larger sized follicles (possibly dominant follicles; 5–9 and 10–14 mm in diameter) decreased on day 3, may be due to ovulation after the luteinizing hormone (LH) surge, associated with hCG administration, resulting in the development of new, small follicles by day 5. Overall, our results indicate that various hCG administration methods do not significantly affect ovarian follicular development but may affect the size and category of follicular numbers.

One advantage of using hCG instead of GnRH to induce ovulation is that the actions of hCG are not affected by *in vivo* P4 levels. Conversely, the ovulatory response to GnRH may vary significantly, depending on the endocrine milieu at the time of injection, because the magnitude of the LH surge is dependent upon the circulating concentrations of P4 (13). Our results suggest that the hCG administration method (dose and/or pattern) in cows with COFs may affect the ovarian response, and thus the therapeutic efficacy. Recently, a treatment protocol, based on the sequential administration of GnRH, PGF_2α_, and GnRH (GPG protocol), was developed as a therapeutic strategy for ovarian cysts (16, 17). Our results regarding follicular development of cows with COF suggest that repeated, low-dose hCG administration, instead of one single GnRH injection, may be a practical alternative treatment for COFs, especially considering that repeated low-dose hCG administration was associated with the recruitment of a significantly larger number of follicles < 4 and 5–9 mm in diameter.

In conclusion, our results suggest that low dose and repeated administration of hCG may be an effective alternative therapy for cows with refractory COFs. Ovarian follicular recruitment and further development were affected by the different hCG dosages in the study. However, ovulatory response and CL formation were not different among the various hCG treated groups. Further research under field conditions and involving a large number of cows and measurement of anti-hCG antibodies after hCG administration, are warranted.

## Author contributions

TeO and TaO conceived and designed the experiments. TeO, MiT, CK, MaT and TaO performed the experiments. TeO, MiT, MW, PV, FT and TaO contributed to write draft preparation. All authors contributed to review the paper.

### Conflict of interest statement

The authors declare that the research was conducted in the absence of any commercial or financial relationships that could be construed as a potential conflict of interest.
